# Supplementary material for: Response of chemical and biochemical soil properties to the spreading of biochar-based treated olive mill wastewater
Source: Heliyon. 2023 Nov 25;9(12):e22894. doi: 10.1016/j.heliyon.2023.e22894 (PMC10730756; doi:10.1016/j.heliyon.2023.e22894)
Supplement: Multimedia component 1 [file mmc1.docx]

**Supplementary Material**

**Biochar-based treatment of olive mill wastewater and effect of their spreading on chemical and biochemical soil properties**

Giuseppe Di Rauso Simeone^*^, Giuseppina Scala, Marcello Scarpato, Maria A. Rao

Department of Agriculture Sciences, University of Naples Federico II, via Università 100, 80055, Portici, Italy

*Corresponding author: Giuseppe Di Rauso Simeone e-mail: [giuseppe.dirausosimeone@unina.it](mailto:giuseppe.dirausosimeone@unina.it)

Pages: 6

Tables: 1

Figures: 3

**Content**

Table S1. Physical and chemical properties of soil.

Figure S1. PVC tube used in the lab-scale experiment.

Figure S2. Chromatogram of OMW at 279 nm. Numbers correspond to 1) gallic acid; 2) 2,6-hydroxybenzoic acid; 3) protocatechuic acid; 4) caffeic acid; 5) 2-hydroxybenzoic acid; 6) catechol; 7) syringic acid (internal standard).

Figure S3. Adsorption of a) 0.5 mg mL^-1^ , b) 1 mg mL^-1^ and c) 1.5 mg mL^-1^ catechol on BP 5%, 10% and 15%.

Table S1. Physical and chemical properties of soil.

| Properties | Value |
| --- | --- |
| Sand (g kg^-1^) | 879 ± 42 |
| Lime (g kg^-1^) | 93 ± 47 |
| Clay (g kg^-1^) | 27 ± 5 |
| pH (in H_2_O) | 7.90 ± 0.06 |
| EC (dS m^-1^) | 0.1 ± 0.03 |
| Limestone (g kg^-1^) | 6.2 ± 0.4 |
| CEC (cmol(+) kg^-1^) | 15 ± 1 |
| TOC (g kg^-1^) | 12 ± 1 |
| O.M. % | 2 ± 0.1 |
| Total N (g kg^-1^) | 1.22 ± 0.02 |
| C/N | 10 ± 0.7 |
| P_2_O_5_ (mg kg^-1^) | 46 ± 1 |


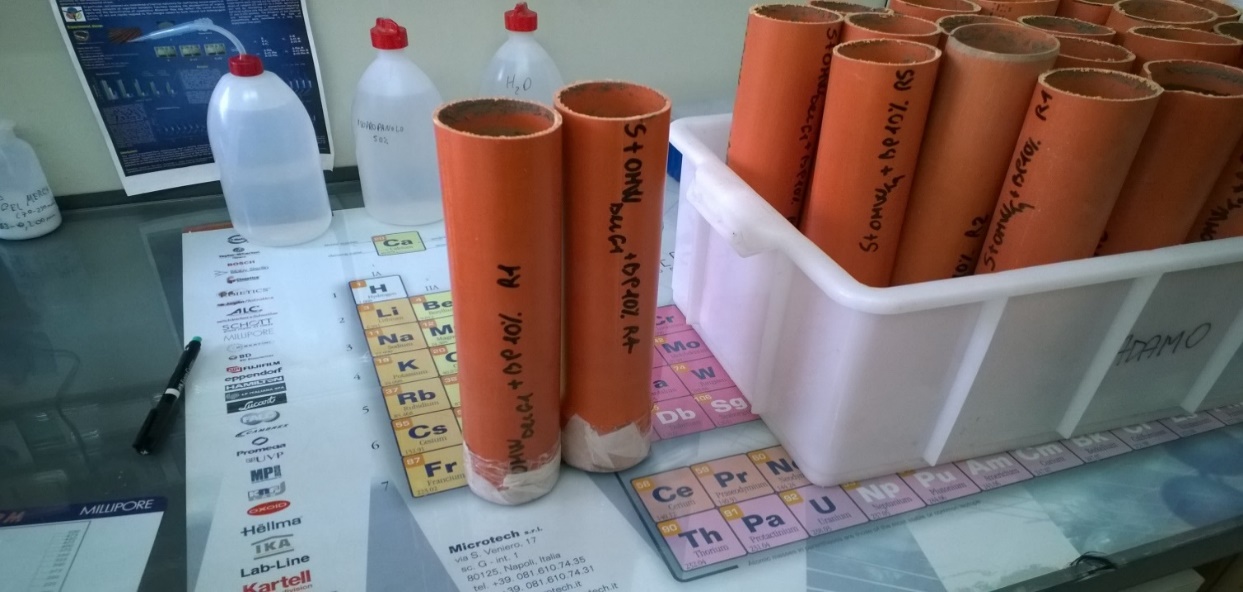


Figure S1. PVC tube used in the lab-scale experiment.


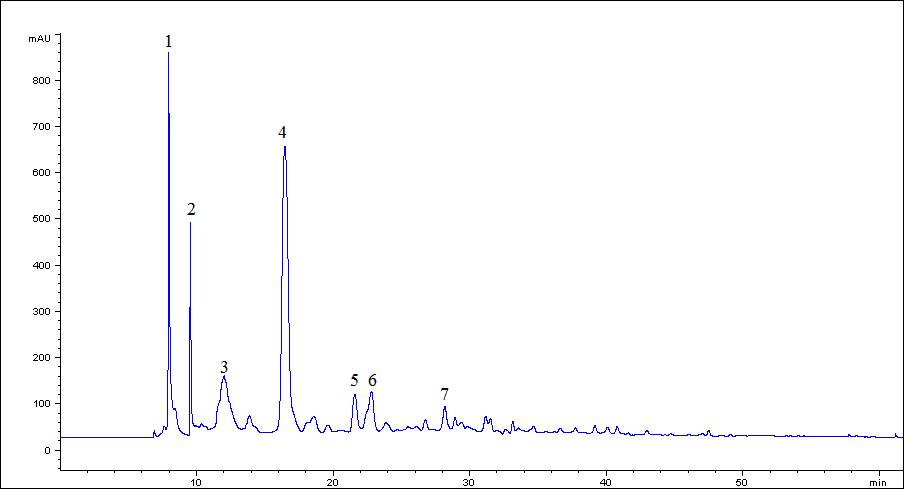


Figure S2. Chromatogram of OMW at 279 nm. Numbers correspond to 1) gallic acid; 2) 2,6-hydroxybenzoic acid; 3) protocatechuic acid; 4) caffeic acid; 5) 2-hydroxybenzoic acid; 6) catechol; 7) syringic acid (internal standard).


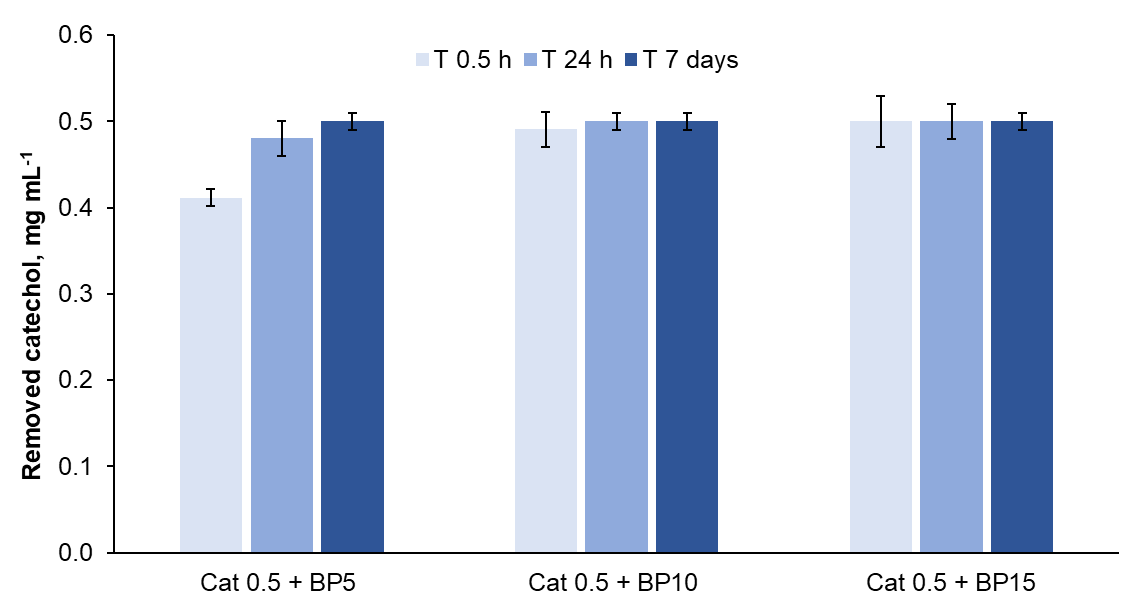


a


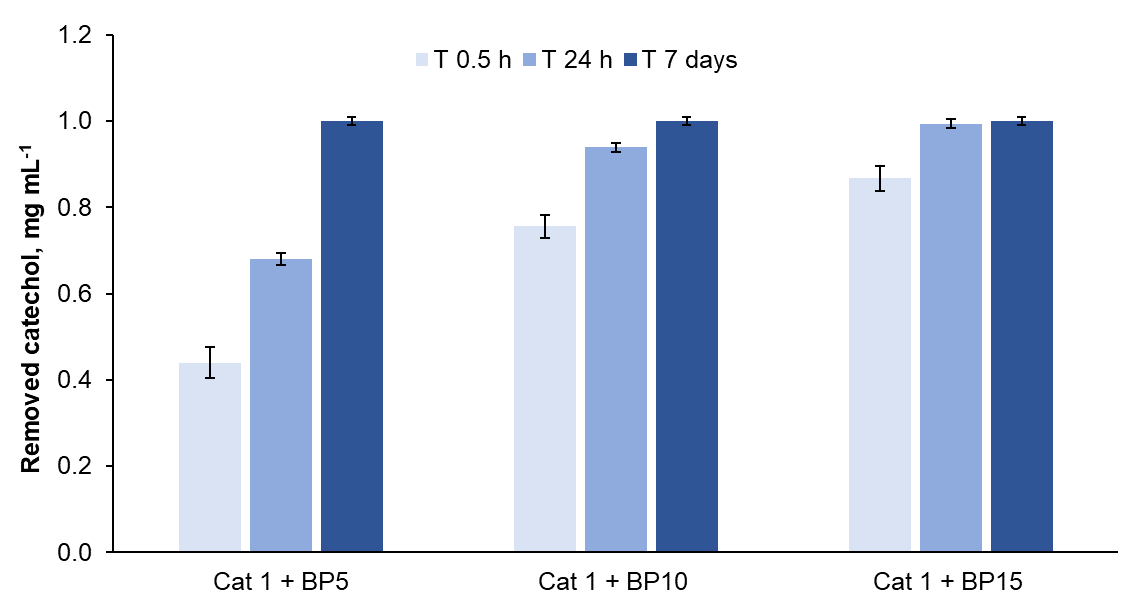


b


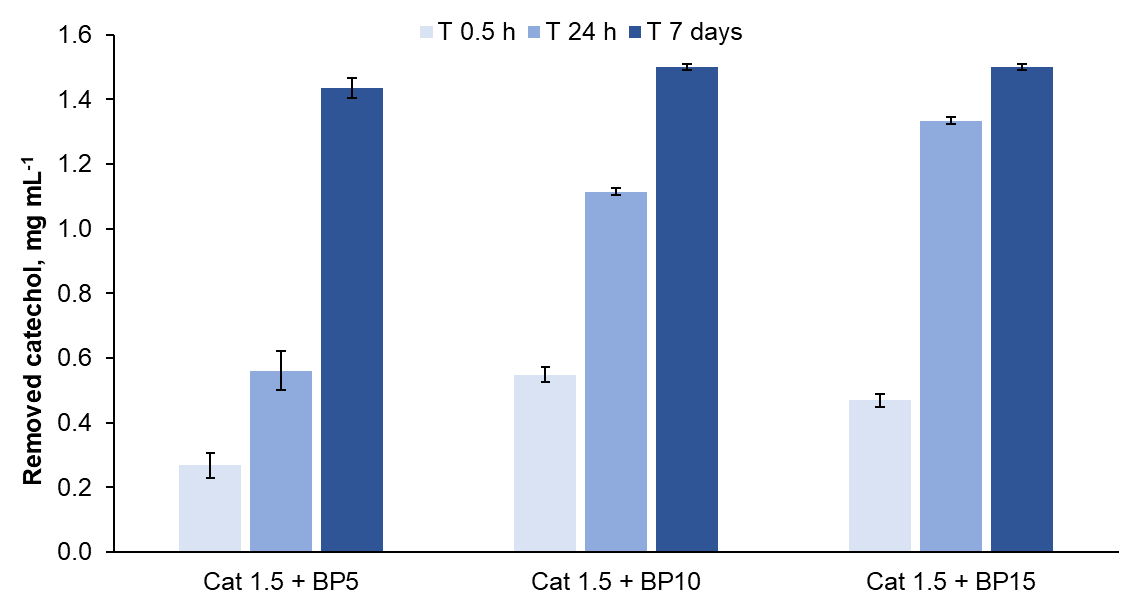


c

Figure S3. Removal of catechol in solutions prepared at 0.5 mg ml^-1^ (a), 1 mg ml^-1^ (b), and 1.5 mg ml^-1^ concentration by 5%, 10% and 15% BP.
